# Psychiatry

**Published:** 1905-11-25

**Authors:** 


### PSYCHIATRY.

**Mental Depression and Melancholia.**—Dr. Arthur Townsend,[1] Senior Assistant Physician at Barnwood House Asylum, refers to the views held at the present day which regards toxic action as the basis of depression and melancholia. According to this view, the depressive form of insanity is not the primary condition, but is the reaction of the brain to toxins which affect it, as would those of tuberculosis (respiratory) or other toxins (of the alimentary canal). The older view, that mental diseases were in some mysterious way "primary," an outcome of metaphysical speculation, which Griesinger said "had long darkened the pathways of psychiatry," may be regarded as obsolete; there are no primary insanities. Auto-intoxication from the alimentary canal is increasingly being recognised as being a source of, if not a factor in, the production of certain insanities of the type of katonia and precocious dementia, now common in our youths and adolescents of both sexes. During the past two years Dr. Townsend examined, as a symptom, the urine to ascertain the presence of indoxyl (or its by-product, indican) in all cases of acute insanity, for the reason that indoxyl in excess indicates abnormal putrefactive processes in the gastro-intestinal tract. Jaffe's test, namely, to mix the urine with an equal quantity of strong HCl. acid, was used, and a minute amount of Ca. hypochlorite added, great care being taken to avoid excess. Indigo was formed and separated in the mixture, which, on being now shaken up with chloroform and allowed to settle, showed the deep blue colour of indigo in the chloroform which separates out at the base of the test. A faint blue appears, says Dr. Townsend, in normal urine. A brilliant bright blue, or a very deep blue, only appears in morbid states, such readily recognisable excess of colour indicating the abnormal putrefactive process taking place with the formation of toxins in the alimentary canal which are poisonous to the animal economy. Excessive production of indoxyl was found by Dr. Townsend in cases of both so-called "mania" and "melancholia." "Indol is invariably excreted in excess in depressed states, while in maniacal conditions it is only excreted in normal, or less than normal, amount." In two exceptional cases of acute mania out of 13, however, a moderate amount of indoxyl was present; these will be referred to later. The cases studied were found to fall into three groups: (*a*) Indoxyl was present in large excess in acute melancholia of the confusional type in two women, in melancholia agitata in two others, in confusional melancholia passing into stupor in a fifth, in melancholia associated with tuberculosis in the next two cases (also females), and finally in recurrent acute melancholia in a woman, which followed a temporary attack of acute excitement—a total of eight cases. (*b*) In other cases of rather slight depressive melancholia a moderate and distinct excess of the same was detected. This, considering the large number of cases systematically and carefully studied —a full total of 16 cases—showed, concludes Dr. Townsend, "that the putrefactive processes resulting in the formation of toxins is primary and causative: it is evident that depth of the mental disorder bears proportion to the degree of toxæmia (intoxication)." It is a striking clinical fact that 11 of the 16 were the subjects of hereditary tendencies, and showed various symptoms of cerebral and mental instability, favouring the supposition that such individuals are more prone to the effects of a particular toxæmia than healthy individuals. "Some experiments which I made favour these views," adds the author. (*c*) Maniacal cases, on the other hand, showed only the normal traces of indoxyl, or even less, in their urine. Thus, while in 13 cases of acute mania, including one of *folie circulaire*, there was the normal of indoxyl, or even a smaller trace, in 11 cases. A nearly normal amount existed in the circular case and in a special case of puerperal acute mania. The 16 cases of depressive melancholia all exhibited very large, or moderately large, excess. Mental recovery among the cases recorded was preceded in each case by the reduction to normal of the amount of indoxyl excreted, a valuable prognostic symptom. What the causes were, however, which facilitated or caused the free auto-intoxication which occurred in these cases remains, concludes Dr. Townsend, a problem for further inquiry.

[1] Jour. of Ment. Sci., January, 1905.

(*To be continued.*)

